# Papaya Leaf Extracts as Potential Dengue Treatment: An In-Silico Study

**DOI:** 10.3390/ijms232012310

**Published:** 2022-10-14

**Authors:** Ayesh Madushanka, Niraj Verma, Marek Freindorf, Elfi Kraka

**Affiliations:** Department of Chemistry, Southern Methodist University, 3215 Daniel Avenue, P.O. Box 750314, Dallas, TX 75275, USA

**Keywords:** dengue fever, papaya leaf extract, ADME, QM/MM, local mode analysis

## Abstract

Dengue fever (DF), dengue hemorrhagic fever (DHF), and dengue shock syndrome (DSS) cause serious public health problems, with nearly 390 million people affected and 20,000 deaths per year in tropical and subtropical countries. Despite numerous attempts, no antiviral drug or vaccine is currently available to combat the manifestation. The challenge of discovering an efficient vaccine is enhanced by the surplus presence of efficient vectors and drug resistance from the virus. For centuries, papaya (Carica papaya) extracts have been traditionally used to treat DF, DHF, and DSS. In the present study, we systematically investigated seven compounds isolated from papaya leaf extract with regard to their potential as inhibitors for non-structural (NS) proteins, NS3 and NS5, which play a crucial role in viral RNA replication. The computational tools applied stretched across classical molecular docking, molecular dynamics (MD) simulations and SwissADME used to calculate binding affinities; binding free energies; Absorption, Distribution, Metabolism, and Excretion (ADME); and drug-likeness properties, thus, identifying Kaempferol, Chlorogenic acid, and Quercetin as potential candidates, with Kaempferol and Quercetin scoring best. Therefore, for the Kaempferol and Quercetin complexes, hybrid quantum mechanical/molecular mechanical (QM/MM) geometry and frequency calculations were performed, followed by the local mode analysis developed in our group to quantify Kaempferol-NS and Quercetin-NS hydrogen bonding. Given the non-toxic nature and the wide availability of the Kaempferol and Quercetin papaya extract in almost all of the susceptible regions, and our results showing high NS3 and NS5 binding affinities and energies, strong hydrogen bonding with both NS3 and NS5, and excellent ADME properties, we suggest Kaempferol and Quercetin as a strong NS3 and NS5 inhibitor to be further investigated in vitro.

## 1. Introduction

Dengue is an acute mosquito-borne disease transmitted by *Aedes aegypti* and *Aedes albopictus* [1], and is rapidly spreading in more than 128 tropical and sub-tropical countries, resulting in an at-risk population of over four billion. The dengue virus (DENV) infects approximately 390 million people yearly, and is responsible for more than 20,000 deaths [2,3,4]. DENV belongs to the Flaviviridae family and *flavivirus* genus, which also includes the hepatitis C virus, the yellow fever virus, the Japanese encephalitis virus, the West Nile virus, the tick-borne encephalitis virus, and the Zika virus [5]. There are four recognizable serotypes of DENV 1–4, with 65–70% of the sequence conserved in each serotype. DENV 2 serotype is the most prevalent one [6,7].

Patients who are hospitalized for dengue fever (DF)—in particular, those with dengue hemorrhagic fever (DHF) or dengue shock syndrome (DSS)—often die from the disease [8]. Despite the release of the FDA-approved vaccine Dengvaxia, which has been approved in 19 countries [9,10], there is no generally accepted anti-viral agent for the DENV virus [11]. Further, Dengvaxia is only recommended for those previously infected with the dengue virus and children who are 9–16 years old. Children without a previous dengue infection are at an increased risk for severe dengue disease and hospitalization if they get dengue after they are vaccinated with Dengvaxia [10].

**Figure 1:**
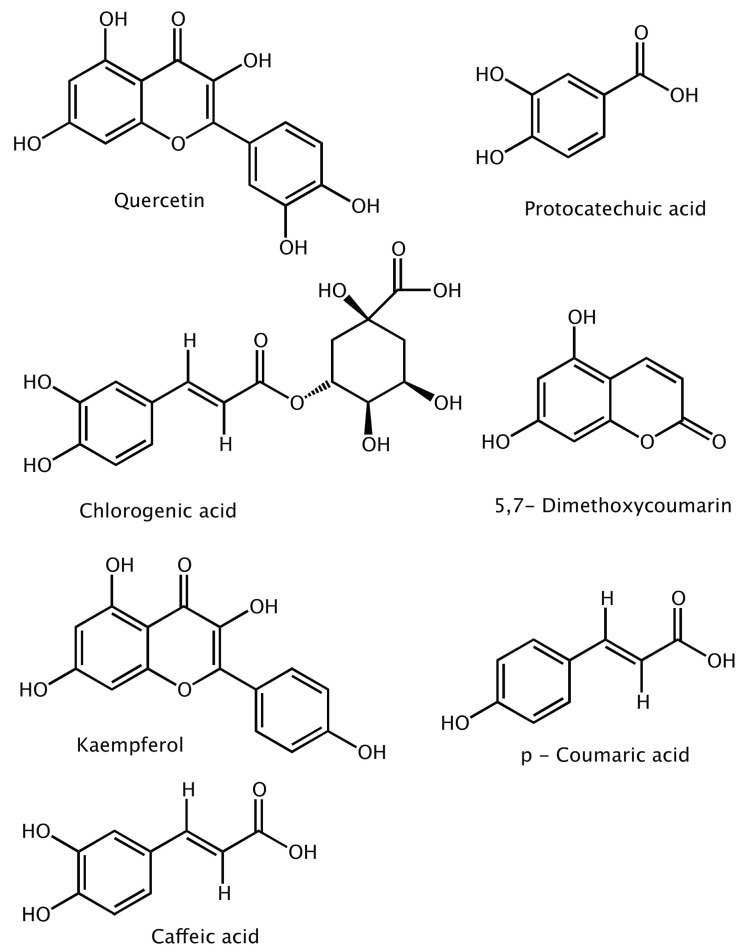
The seven papaya leaf extracts characterized by Canini et al. [36].

Dengue is linked to thrombocytopenia, which is a specific feature of dengue disease [12]. A thrombocyte count lower than 100,000 cell/mm^3^ leads to DHF. The virus can bind to the human platelet with the presence of antibodies which are produced after a dengue infection, Refs. [13,14,15] or through an immune response [16,17,18]. Several studies have concluded that papaya leaves can significantly increase platelet count [19,20,21,22,23,24,25]. Carica papaya (CP), a member of the family Caricaceae, grows in the West Indies, the Philippines, Sri Lanka, India, Bangladesh, Malaysia and other countries in tropical America. Papaya leaf extracts (PLE) have become popular as a traditional remedy for various ailments, including the Human Immunodeficiency Virus (HIV) [26], wound healing [27], anti-sickling [28], chronic kidney diseases [29], anti-bacterial treatment, reversible male infertility [30], and dengue [31]. While Carpaine in PLE is the major active compound that contributes to anti-thrombocytopenic activity [32,33], other phenolic compounds might also be acting as antivirals in synergy, as suggested by Sharma et al. [34] via in vivo studies, showing that PLE can be active as an antiviral towards DENV [12,35]. The gas chromatography-mass spectrometry (GC-MS) analysis of PLE carried out by Canini et al. [36] extracted seven phenolic compounds (Figure 1). Molecular docking analysis provided by Senthilvel et al. [31] suggests the efficacy of these compounds as antiviral agents toward DENV 2 [37].

The non-structural (NS) proteins, NS3 and NS5, are the major targets for new anti-dengue drug therapies due to their involvement in RNA replication; a process that plays a significant role in the spread of infection. The NS proteins expressed in the host cell reshape the inner organization and maturation of the virus, replicate viral RNA, and even help evade the immune system [38]. The NS3 protease is a multi-functional protein of 618 amino acids that can act as serine protease as well as RNA helicase and RTPase/NTPase [2]. The association of RNA is crucial for the maturation of the viral particles, creating NS3 as a promising anti-dengue target for drug development [39,40,41]. The other key player in DENV is the NS5 proteome, which is the largest and most conserved non-structural protein, with 900 amino acid residues and approximately 67–85% of the amino acid sequence conserved between the four serotypes [38]. The RNA polymerase domain plays a crucial role in the viral replication process, which generates negative polarity RNA from the positive viral RNA template [42]. The generated negative RNA acts as a starting template for a more positive RNA synthesis that translates to other virus proteins [43]. NS5 can also interfere with the host’s immune system and deregulate the immune response using a signal transducer and the activator transcription 2 (STAT2) protein, or by modulating RNA splicing within the host cell [44,45].

The major goals of the present study were (i) to investigate the inhibitory ability of the seven phenolic compounds shown in Figure 1 for the two major non-structural proteins—NS3 and NS5—of DENV 2 responsible for viral replication, which were originally extracted from papaya leaves by Canini et al. [36]; (ii) to explore which of the seven phenolic compounds can bind with NS3 and NS5; (iii) to determine the stability of the protein–ligand complexes formed, particularly the strength of these interactions; and (iv) to exploit their ADME properties. The overarching goal was to develop a computational protocol for this purpose and to explore if the results obtained in this study can open a new chapter for the design of novel selective inhibitors for the dengue virus and also other flavivirus genera.

## 2. Methodology

### 2.1. Ligands and the NS3 and NS5 Protein Preparation

The X-ray crystal structures of NS3 (PDB ID-2FOM) [46] and NS5 (PDB ID-5ZQK) [47] for DENV2 were obtained from the RCSB protein data bank [48]. The NS3 and NS5 proteins were prepared with the UCSF Chimera software package, Ref. [49], where the inhibitor molecules and water were removed from the binding pocket and the NS5 dimeric protein structure was converted into the monomeric structure. The seven CP-leaf extracted compounds were accessed from PubChem [50] and the 3-Dimensional (3D) structures were prepared on the UCSF Chimera.

### 2.2. Classical Docking

Molecular docking is a powerful technique for studying potential ligand–receptor interactions. Most often, it is based on molecular mechanics methodologies, i.e., classical docking. These methods are faster than hybrid quantum chemical/molecular mechanic (QM/MM) methodologies, Refs. [51,52]. However, they provide more qualitative results on potential ligand–receptor interactions. An essential part of each docking program is the scoring functions used for sampling and the prediction of binding energies [53]. In this study, we used the molecular docking software, Smina [54], which leverages scores through an empirical and knowledge-based approach based on the CSAR-NRC HiQ 2010 data set [54]. The Open Babel [55] package was employed to convert PDB (Protein Data Bank) coordinates into the PDBQT (Protein Data Bank, Partial Charge (Q), and Atom Type (T)) format which includes partial charges (‘Q’) and AutoDock 4 (AD4) atom types (‘T’). The binding pocket coordinates of the NS3 (GLY151, GLY153, SER135, TYR150, ASP129, and PHE130) and NS5 (HIS110, GLU111, GLU83, GLU86, TRP87, ASP146, ILE147, VAL132, and ASP131) proteins were adapted from Erbel et al. [46] and El Sahili et al. [47], respectively. The three best docking CP-leaf extracted compounds (out of the seven) on the NS3 protein, as well as the NS5 structures, were then subjected to MD simulation. Ligand interaction diagrams were prepared using the ligand preparation wizard from Schrödinger, Maestro [56].

### 2.3. Molecular Dynamics (MD) Simulation

The basic MD engine of AMBER [57], *Sander*, was employed for the MD simulation, and the FF14SB [58] version of the AMBER force field was used to describe both proteins. To generate the ligand topologies, General Amber Force Field (GAFF) was used with ANTECHAMBER, [59] which generates atomic partial charges utilizing the Restrained Electrostatic Potential (RESP). The *tleap* module of AMBER16 [60] was utilized to add hydrogens and other missing atoms to the experimental structure, as well as Na${}^{+}$ and Cl${}^{-}$- counter ions for the neutralization of the NS3 and NS5 proteins. The NS3 and NS5 proteins with their ligands were then suspended in a 10 Å and 12 Å box of TIP3P [61] water molecules, respectively.

AMBER minimization was carried out for 2000 steps using the steepest descent minimization and conjugate gradient minimization. All systems were then slowly heated from 0 K to 300 K for 50 ps, and another 50 ps with a potential harmonic restraint of 25 kcal/mol. MD simulations of both unbound proteins and the protein–ligand complexes were conducted for 50 ns. The SHAKE algorithm [62] was utilized to constrict hydrogen bonding (HB). The step size for each simulation was 2 fs and the temperature was kept constant at 300 K. In addition to the temperature, the total pressure of the system was also kept constant at 1 bar, using the *Berendsen barostat* to mimic [63] the isobaric–isothermal ensemble (NPT).

### 2.4. Post-MD Simulation Trajectory Analysis and Data Analysis

The CPPTRAJ [64] module in the AMBER package was used to generate Root Mean Square Deviation (RMSD) and Root Mean Square Fluctuation (RMSF) parameters from the MD trajectory files. All raw data plots were generated using the DataGraph software [65].

### 2.5. Binding-Free-Energy Calculation

Binding free energies were calculated by averaging the snapshots extracted from the MD simulation on the molecular mechanics energies combined with the generalized Born and surface area continuum solvation (MM/GBSA) method in AMBER package [66]. Binding free energies were calculated by subtracting the total of free energies of the unbound receptor and ligand from the free energy of the bound complex, according to Equation (1):

(1) $$\begin{array}{cc} \Delta G_{binding,solvated} & =\Delta G_{complex,solvated}-\left[\Delta G_{receptor,solvated}+\Delta G_{ligand,solvated}\right]\\ \Delta G_{solvated} & =E_{gas}+\Delta G_{solvation}-TS_{solute} \end{array}$$

Gas-phase energies ($E_{gas}$) were extracted from the force field, whereas the solvation free energies (Δ$G_{solvation}$) were calculated using an implicit solvent model. The entropic contribution (S) was estimated using standard protocols [67]. All the calculations were triplicated and the average value was taken as the final energy.

### 2.6. Prediction of Protein Targets and Pharmacokinetic Properties

The SwissTargetPrediction software package [68] was employed to determine therapeutic targets for the selected PLE compounds. This was useful to understand more about their general bioactivity and to rationalize possible side effects. The SwissADME [69] program was utilized to predict the pharmacokinetic properties of these compounds. Lipophilicity calculations were carried out using the GBSA approach [70], and the BOILED-Egg [71] method was employed to predict the blood–brain barrier (BBB) and gastrointestinal (GI) penetration.

### 2.7. Local Mode Analysis

In order to quantify HBs between ligand (Kaempferol and Quercetin) and the active site pockets of NS3 and NS5, respectively, we performed the local mode analysis (LMA) based on QM/MM vibrational calculations of the corresponding normal modes. LMA was originally developed by Konkoli and Cremer [72,73,74,75] and has recently been described by a comprehensive review article [76]; therefore, in the following, only some of the highlights of LMA are summarized.

Information on the electronic structure and bonding of a molecule is encoded in the normal vibrational modes. However, normal vibrational modes are generally delocalized as a result of kinematic and electronic coupling [77,78,79]. A particular normal stretching mode between the two atoms of interest can couple to other stretching, bending or torsional normal modes, inhibiting the direct correlation between the normal stretching frequency or associated normal mode force constant with bond strength, as well as the comparison between stretching modes in the related molecules. The electronic coupling is generally eliminated during a standard frequency calculation following the Wilson GF–formalism [77,80,81,82] by transforming from Cartesian coordinates X to normal mode coordinates Q and related normal modes, resulting in a diagonal force constant matrix $K^{Q}$.

However, this does not eliminate the kinematic (mass) coupling which often has been overlooked. Konkoli and Cremer solved this problem via solving mass-decoupled Euler–Lagrange equations [72,73,74,75,77] by setting all atomic masses to zero except those of the molecular fragment (e.g., bond, angle, or dihedral, etc.) carrying out the localized vibration under consideration. The local mode $a_{\mu }$ of a molecular fragment associated with an internal coordinate $q_{\mu }$ is given then by

(2) $$\begin{array}{c} a_{\mu }=\frac{K^{-1}d_{\mu }^{†}}{d_{\mu }K^{-1}d_{\mu }^{†}} \end{array}$$

where $d_{\mu }$ corresponds to a row vector of the normal mode matrix D in internal coordinates $q_{\mu }$ [77].

The local mode force constant $k_{\mu }^{a}$ corresponding to local mode $a_{\mu }$ is obtained by

(3) $$\begin{array}{c} k_{\mu }^{a}=a_{\mu }^{†}Ka_{\mu } \end{array}$$

Other local mode properties such as local mode frequencies, masses, and intensities can be defined accordingly [76].

In particular, the local mode force constants k${}^{a}$ have qualified as a quantitative measure of bond strength for both covalent bonds [83,84,85,86,87,88,89] and weak chemical interactions, such as halogen bonds, refs. [90,91,92,93,94,95], chalcogen bonds, refs. [96,97,98], pnicogen bonds, refs. [99,100,101] tetrel bonds, ref. [102], and hydrogen bonds [103,104,105,106,107,108,109,110,111]. It is convenient to base the comparison of the bond strength for the set of molecules on a chemically more-prevalent bond strength order (BSO n) rather than on a comparison of local force constant values. Both are connected via a power relationship according to the generalized Badger rule derived by Cremer and co-workers [85]: BSO n = A ($k^{a}$)${}^{B}$. The constants A and B can be determined from two reference compounds with known BSO n values and the requirement that for a zero force constant, the BSO n is zero. For HBs, we generally use the FH bond as references in the FH molecule with BSO n = 1 and the FH bond in the [F⋯H⋯F]${}^{-}$ anion with BSO n = 0.5 [103,106,109,111]. In this work we used HF with (k${}^{a}$ = 9.935 mDyn/Å) and F${}_{2}$H${}^{-}$ (k${}^{a}$ = 1.204 mDyn/Å) as a reference (ωB97X-D/6-31G(d,p) level of theory) which yielded the values A = 0.4704 and B = 0.3284. QM/MM calculations were performed with Gaussian, ref. [112], and LMA was carried out with the LModeA program package [113]. Computational details of the QM/MM protocol applied in this work can be found in the Supporting Information.

## 3. Results and Discussion

**Figure 2:**
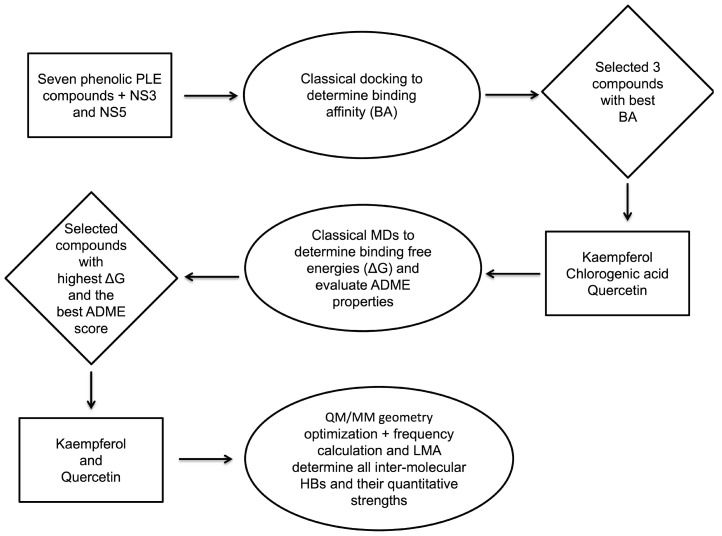
Flow chart of the dengue study.

Figure 2 describes the overall workflow protocol developed for our dengue study. First, a docking analysis was carried out to determine the best binding affinities (BA) of the compounds for both the NS3 and NS5 non-structural proteins. Then, the three compounds (Kaempferol, Quercetin, and Chlorogenic acid) with the highest BAs were selected for further examinations. MD calculations were carried out for the three compounds for 50 ns to identify the stability of the protein–ligand complexes in an aqueous environment. Then, MM/GBSA binding free energies were calculated based on the MD trajectories. We further explored their ADME as well as pharmacokinetic properties, e.g., their drug-likeness via Lipinski’s rule-of-five test [114,115], complemented by potential target analysis to identify the best candidates. However, Chlorogenic acid showed negative results for drug-likeness and lead-likeness tests with one Pan-Assay Interference compound (PAINS) alert. Therefore, we employed unconstrained QM/MM geometry optimizations and frequency calculations for the Kaempferol-NS and Quercetin-NS complexes, complemented with LMA to determine all possible HBs between the protein-binding pockets and ligands as well as their quantitative strengths; these HBs play a key role for the complex stability and, as such, for the inhibitory strength of the ligand.

### 3.1. Classical Docking

**Figure 3:**
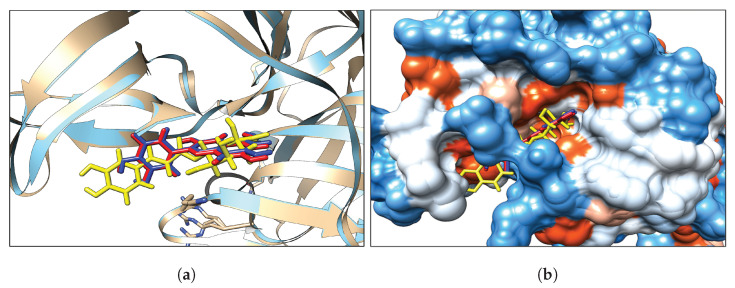
(**a**) NS3 protein Ribbon, (**b**) NS3 hydrophobicity surface with colors ranging from dodger blue for the most hydrophilic, white for neutral (i.e., 0.0 value), to orange red for the most hydrophobic. (Blue–Quercetin, red–Kaempferol, and yellow–Chlorogenic acid).

**Figure 4:**
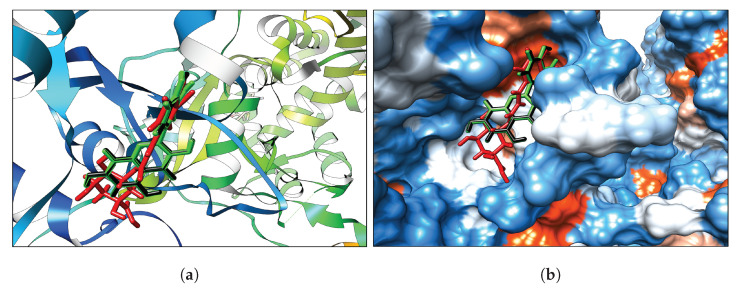
(**a**) NS5 protein Ribbon, (**b**) NS5 hydrophobicity surface with colors ranging from dodger blue for the most hydrophilic, white for neutral (i.e., 0.0 value), to orange red for the most hydrophobic. (Green–Quercetin, black–Kaempferol, and red–Chlorogenic acid).

The binding sites of the NS3 and NS5 proteins are orthosteric. NS3 cleaves the viral polyprotein and separates the double-stranded RNA (dsRNA) intermediate during viral RNA amplification. NS5 carries out cap methylation and RNA synthesis activities [116]. Thus, efficient viral replications can be prevented by blocking the binding sites. Therefore, these binding pockets were targeted in the docking study. Table 1 shows the highest binding affinities of the extracted phenolic papaya leaf compounds in the binding site of both NS proteins. Binding affinity deviated between −6.2 kcal/mol to −9.2 kcal/mol for the NS3 protein, and −6.0 kcal/mol to −10.1 kcal/mol for the NS5 protein. Quercetin, Kaempferol, and Chlorogenic acid showed significantly higher binding affinities than the other four compounds, with the NS3 between −9.2 kcal/mol and 8.6 kcal/mol, and −10.1 kcal/mol and −9.2 kcal/mol for the NS5. Caffeic acid exhibited significantly lower binding affinities of −6.8 kcal/mol and −6.5 kcal/mol for NS3 and NS5, respectively, with an energy gap between Chlorogenic and caffeic acid of −1.8 kcal/mol (NS3) and −2.7 kcal/mol (NS5). Quercetin exhibited the highest binding affinity for both NS3 and NS5 proteins. Therefore, Quercetin, Kaempferol, and Chlorogenic acid were selected for further analysis. The binding conformations of Quercetin, Kaempferol, and Chlorogenic acid in both NS proteins were taken as favorable poses for further molecular dynamics simulations and binding-free-energy calculations, as shown in Figure 3 and Figure 4, respectively.

#### Interaction Analysis

**Figure 5:**
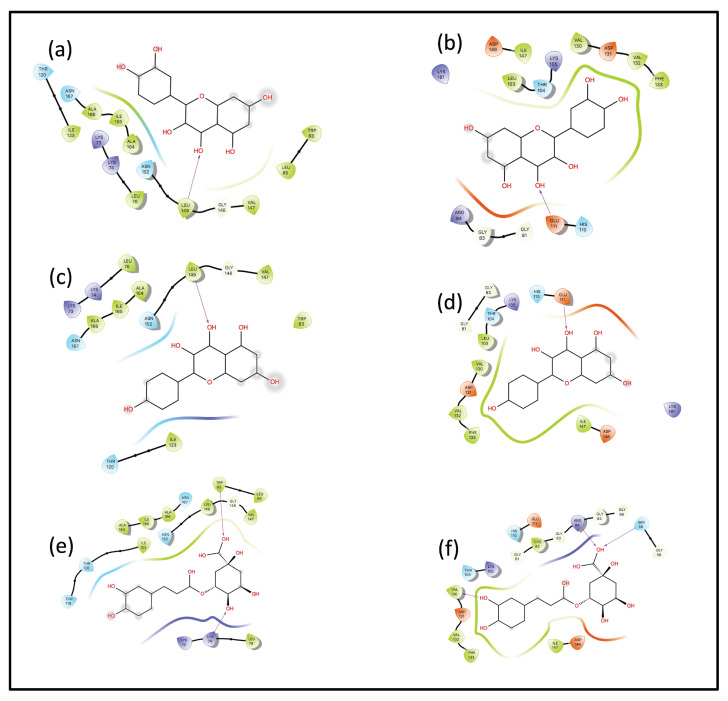
Ligand interaction diagrams for NS3 and NS5 receptors from Schrödinger and Maestro. (**a**) Quercetin + NS3, (**b**) Quercetin + NS5, (**c**) Kaempferol + NS3, (**d**) Kaempferol + NS5, (**e**) Chlorogenic acid + NS3, and (**f**) Chlorogenic acid + NS5. Color codes for residues by orange = charged (negative), purple = charged (positive), white = Glycine, green = hydrophobic, light blue = polar, purple arrow = hydrogen bond.

The interaction diagrams (Figure 5) represent HB patterns between the binding pocket of the protein and the ligand within 4 Å distance. Quercetin and Kaempferol form only one HB with both NS proteins, whereas Chlorogenic acid forms two and three HBs with the NS3 and NS5 proteins, respectively. Leucine-149 binds with one hydroxyl group of both Quercetin and Kaempferol in NS3 and Glutamic acid-111 with the NS5. Lysine-74 and Tryptophan-83 show interactions with two hydroxyl groups of Chlorogenic acid in NS3 and Serine-56, and Arginine-84 and Valine-130 in NS5. The largest number of available hydroxyl groups and non-planar geometry are most likely what leads to the observation that Chlorogenic acid presents the highest number of HBs. However, the QM/MM and LMA section will provide a detailed insight into the HB interactions between binding pockets and ligands.

### 3.2. Binding Free Energy

Molecular docking only determines the geometric fit of small molecules into the active site of a protein; therefore, triple 50 ns long molecular dynamics simulations (MD) were performed and various conformations or snapshots of the solute were extracted from the MD trajectories to calculate the binding free energy of each system utilizing the MM/GBSA method, with the average values taken from the triplication runs. The resulting thermodynamic binding free energies are presented in Table 2, with more negative values reflecting more favorable binding. For both NS3 and NS5, Kaempferol showed the lowest (the most negative value) binding free energy.

Chlorogenic acid displayed the highest number of HBs with NS proteins in the ligand interaction analysis. However, the binding-free-energy analysis confirmed that the number of HBs is not the only factor to determine the stability of the ligand inside the binding pocket, showing the lowest binding free energy of Kaempferol, which was ca. −25 kcal/mol and −26 kcal/mol for NS3 and NS5 proteins, respectively.

#### RMSD and RMSF Analysis

**Figure 6:**
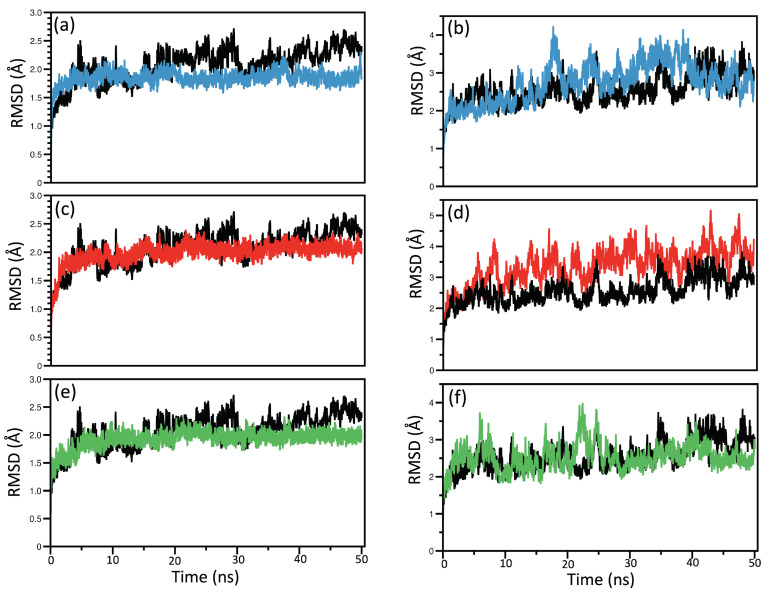
RMSD analysis for NS3 and NS5 receptors. (**a**) Quercetin + NS3, (**b**) Quercetin + NS5, (**c**) Kaempferol + NS3, (**d**) Kaempferol + NS5, (**e**) Chlorogenic acid + NS3, and (**f**) Chlorogenic acid + NS5 by black—unbound protein, blue—Quercetin, rRed—Kaempferol, and green—Chlorogenic acid.

In total, 24 MD simulations were performed as 50 ns triplicates for Quercetin-NS3, Kaempferol-NS3, Chlorogenic acid-NS3, unbound-NS3, Quercetin-NS5, Kaempferol-NS5, Chlorogenic acid-NS5, and unbound-NS5. The RMSD and RMSF analyses were applied to validate protein stability, residues, and ligands composing the protein during the 50 ns MD simulations. The resulting first RMSD plots for the NS proteins are shown in Figure 6. The deviation produced by the protein–ligand complexes during a simulation is a factor for determining their stability, where lower deviations represent a more stable complex. The maximium deviations of the RMSDs less than 2.0 Å correspond to stable structures for ligand–protein systems [117]. RMSDs of the second and third MD simulations are shown in Figures S2 and S4 of the Supporting Information. The NS5 protein complexes and unbound protein deviated only between 2.0 Å to 4.0 Å distance in all three simulations and showed the same behavior. As expected, some fluctuations were visible over the course of the simulations. Although the second and third RMSDs of the NS3 protein showed more distinct deviations from the first simulation run, all simulations deviated only in the 2–4 Å range, reflecting the stability of the ligands inside the binding pocket. Furthermore, all systems obtained equilibration at around 10 ns, and the systems remained stable until 20 ns. In the case of the second simulation, Kaempferol and Chlorogenic acid showed a sudden increase in the deviation so that we carried out another RMSD analysis for the NS3 binding pockets (residues between a 5 Å distance from the ligand). The RMSD analysis of the binding pocket is shown in Figure S3 of the Supporting Information, depicting the stability of the protein–ligand complexes and showing the equilibration after 10 ns, except for Chlorogenic acid—a result which added to the decision of excluding Chlorogenic acid from further QMMM calculations and LMA analyses.

**Figure 7:**
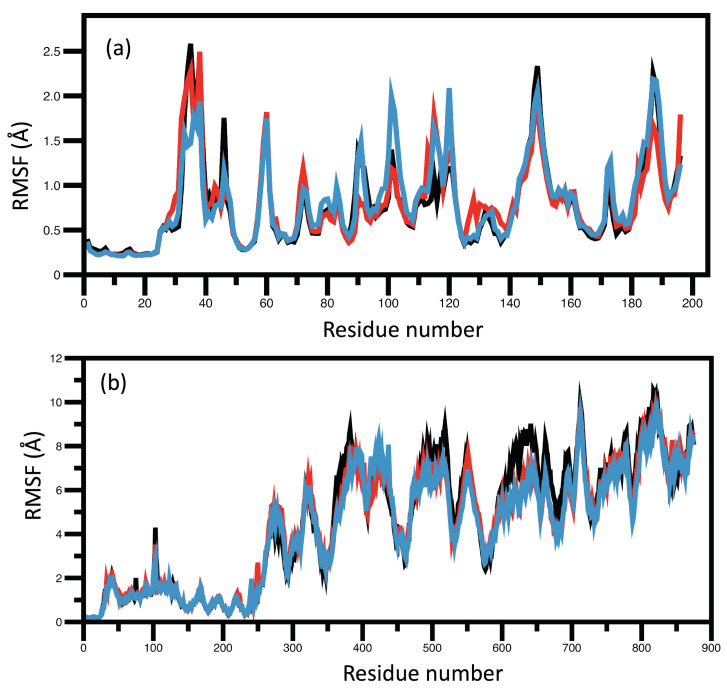
RMSF analysis for NS3 and NS5 receptors. (**a**) RMSF-NS3, (**b**) RMSF-NS5 by black—Quercetin, red—Kaempferol, and blue—Chlorogenic acid.

RMSF plots examine the fluctuations of each amino acid residue with respect to its unbound state; this exhibits the flexibility of the each residue or how much a particular residue moves during the simulation, and can indicate structurally which amino acids in a protein contribute the most to a molecular motion [118]. The RMSF graphs produced in this work are shown in Figure 7. The flexibility of a residue depends on several parameters, including terminal residue and surface loop regions that are highly mobile compared to the protein core. Neighboring residues can also alter the flexibility as well as intermolecular interactions and conformational rearrangements [119]. ASN105, LYS104, LEU31, VAL72, LYS73, LYS74, GLU91, THR118, SER158, GLY159, and ALA160 showed intense fluctuations in NS3. All NS3 fluctuations were inside the binding site, except ASN105, LYS104, LEU31, and GLU91. The NS5 RMSF graph showed significantly higher fluctuations for residues 270 and higher. NS5 comprises two distinct domains called the methyltransferase domain (MTase)—which includes residues 1–263 (molecular weight 30 kDa)—and the RNA-dependent RNA polymerase (RdRP) domain—which includes residues 273–900 (molecular weight kDa). In particular, NS5 residues LYS719, ASP720, GLY721, MET825, ASP826, LYS827, THR828, VAL830, and SER832 showed more fluctuations, with most of them being related to the RdRP binding sites [120]. MTase turned out to be more rigid and RdRP more mobile in agreement with the current literature [121].

### 3.3. Drug-Likeliness and ADME Properties

The SwissADME program predicts ADME-related properties such as pharmacokinetics, molecular weights, bioavailability score, lipophilicity, and water solubility of the active compounds to evaluate their drug potentials. The ADME results showed that the three selected compounds are soluble in both aqueous and lipid environments. Moreover, all three compounds passed the drug-likeness (Lipinski’s rule-of-five) test, with the exception of Chlorogenic acid failing on one rule (more than 5 HB donors; marked as Yes(1) in Table 3). BBB permeation calculated by the BOILED-Egg model revealed no BBB permeability (see Table 3), as expected for a good drug candidate. However, Chlorogenic acid received a low bioavailability score and showed low absorption in the gastrointestinal tract, mainly caused by its higher molecular weight. Furthermore, lead-likeness tests showed that only Kaempferol and Quercetin have potential as drug candidates, whereas Chlorogenic acid was rejected by the lead-likeness test, showing one PAINS alert with catechol_A. Analysis of the inhibition effects of CYP for CYP1A2, CYP2D6, CYP2C9, CYP2C19, and CYP3A4 revealed that Kaempferol and Quercetin can inhibit CYP1A2, CYP2D6, and CYP3A4, but none can be inhibited by Chlorogenic acid. The bioavailability indicates the quantity of the active compound absorbed from the pharmaceutical form that reaches the blood circulation, which depends on several factors such as the quantity absorbed by the intestinal epithelium, drug solubility, gastrointestinal pH, and gastrointestinal transit [122]. Overall, Kaempferol and Quercetin represented high water and lipid solubility, no BBB permeability, good bioavailability score, good absorption in the gastrointestinal tract, and good agreement with Lipinski’s rule-of-five and lead-likeness test.

#### Potential Targets for Kaempferol, Quercetin, and Chlorogenic Acid

**Figure 8:**
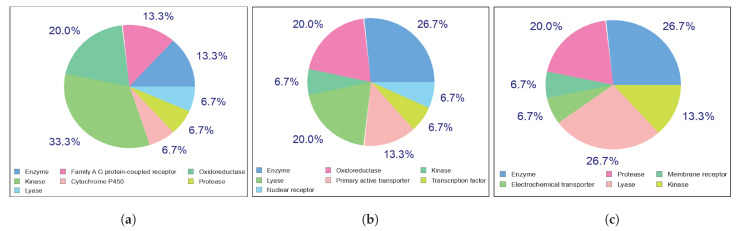
Potential top 15 target classes for (**a**) Quercetin, (**b**) Kaempferol, and (**c**) Chlorogenic acid.

Figure 8 shows the top 15 target classes for selected active compounds. Potential targets can be employed as indicators to investigate bioactivity, possible side effects, off-targets, and the possibility of repurposing therapeutically-relevant compounds. Enzyme, oxidoreductase, and lyase were predicted as the most probable drug targets for Kaempferol, as well as Kinase and oxidoreductase for Quercetin. Furthermore, Kaempferol and Quercetin exhibited 100% binding probability for 16 and 70 possible drug targets, respectively. On the other hand, lyase and protease were selected for Chlorogenic acid. However, our active compounds are natural compounds that are extracted from papaya leaves, and human toxicity is negligible. Overall, Kaempferol and Quercetin may bind with more targets than Chlorogenic acid.

### 3.4. QM/MM and LMA Results

#### 3.4.1. Kaempferol-NS3

**Figure 9:**
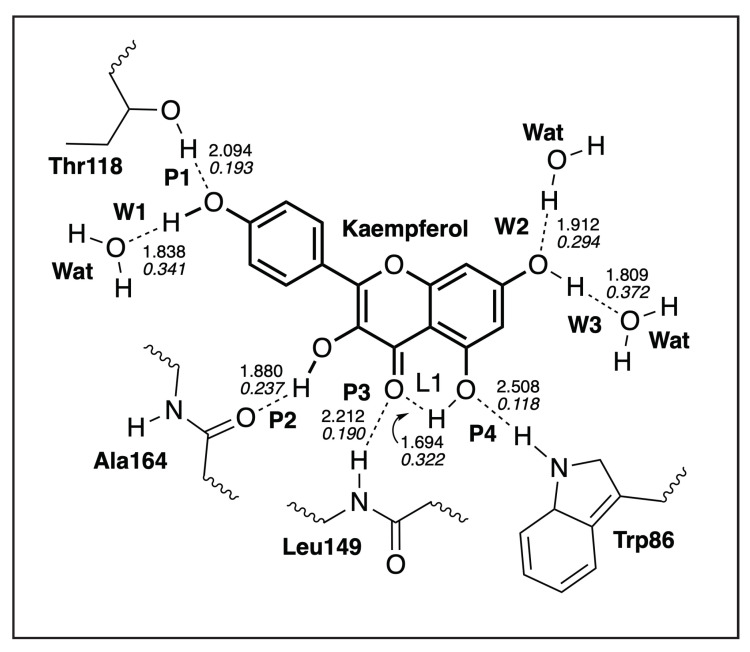
Hydrogen bonds in the Kaempferol-NS3 complex (dashed lines). Bond lengths are shown in the normal font, BSO values are shown in the italic font. ωB97X-D/6-31G(d,p)/AMBER level of theory.

**Figure 10:**
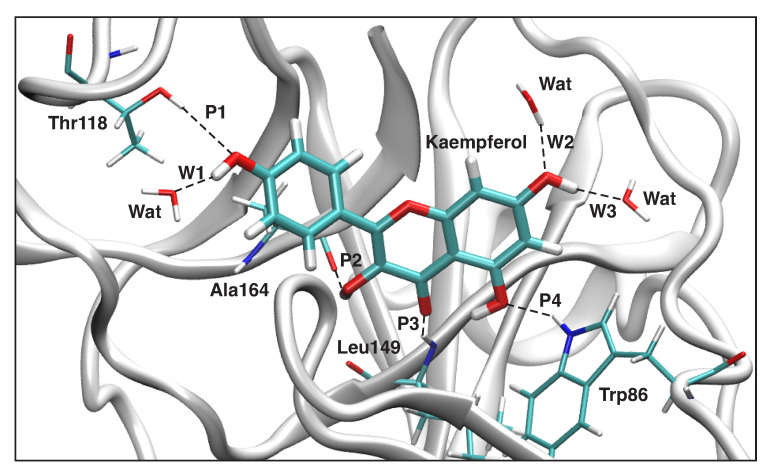
Optimized geometry of the Kaempferol-NS3 complex. The QM part includes Kaempferol, indicated by bold sticks; the MM part includes the NS3 protein, water molecules, and counter ions (for further details, see Supporting Information). There are eight hydrogen bonds (dashed lines) between Kaempferol and the Thr118, Ala164, Leu149, and Trp86 residues (thin sticks) of NS3, and between Kaempferol and the three water molecules in the binding pocket and one intramolecular hydrogen bond; see also Figure 9. (Number of QM atoms: 31, total number of atoms: 3338). ωB97X-D/6-31G(d,p)/AMBER level of theory.

The local mode parameters of the hydrogen bonds between Kaempferol and NS3 from the QM/MM calculations are presented in Table 4, and a sketch of these hydrogen bonds are shown in Figure 9, while Figure 10 presents the picture of the optimal geometry of the ligand in the active site pocket. There are four hydrogen bonds between the ligand and the protein active site pocket—namely, **P1**—involving a hydrogen bond donor from a side chain of Thr118 and oxygen of a hydroxyl group of the ligand, **P2** where the ligand is a hydrogen bond donor to a carbonyl group of a backbone in Ala164, **P3** where an NH group of a backbone in Leu149 donates hydrogen to oxygen of a carbonyl group in the ligand, and **P4** where a hydrogen bond is between an NH group in a side chain of Trp86 and oxygen of a hydroxyl group of the ligand.

There are also three hydrogen bonds between the ligand and water molecules—namely, **W1**, where the ligand is a hydrogen bond donor, and two additional hydrogen bonds with water, where a hydroxyl group of the ligand acts as a hydrogen bond acceptor (**W2**) and a hydrogen bond donor (**W3**). The ligand additionally forms an intramolecular hydrogen bond between hydroxyl and carbonyl groups (**L1**). Table 4 also includes hydrogen bond parameters of a water dimer, calculated in the gas phase **WD**. According to Table 4, the strongest hydrogen bonds are formed between water molecules, which accept a hydrogen atom from a hydroxyl group of the ligand (BSO = 0.372 and d = 1.809 Å for **W3**; BSO = 0.341 and d = 1.838 Å for **W1**). Among the hydrogen bonds between the ligand and the active site pocket, the strongest hydrogen bond **P2** is formed between the backbone of Ala164, which accepts a hydrogen atom from the hydroxyl group of the ligand (BSO = 0.237 and d = 1.880 Å). For the other two hydrogen bonds, **P1** and **P3**, the ligand is a hydrogen bond acceptor, forming a relatively weaker hydrogen bond interaction (BSO = 0.193 and d = 2.094 Å for **P1**; BSO = 0.190 and d = 2.212Å for **P3**). According to Table 4, the intramolecular hydrogen bond **L1** is relatively strong (BSO = 0.322 and d = 1.694 Å), which preserves to form a hydrogen bond between the hydroxyl group involved in **L1**, and side chains or backbones of the protein active site pocket. The reference hydrogen bond of a water dimer in the gas phase **WD** has a medium strength (BSO = 0.288 and d = 1.926 Å) among the investigated hydrogen bonds in this study. Generally, we can conclude that the strongest hydrogen bond interactions are formed between the ligand acting as a hydrogen bond donor and surrounding water molecules (**W1**) and (**W3**). Among the hydrogen bonds with the protein active site pocket, the strongest hydrogen bond interaction regards the ligand acting also as a hydrogen bond donor (**P2**); however, the strength of the hydrogen bonds with the protein pocket is generally smaller than the strength of the hydrogen bonds involving water molecules.

#### 3.4.2. Kaempferol-NS5

**Figure 11:**
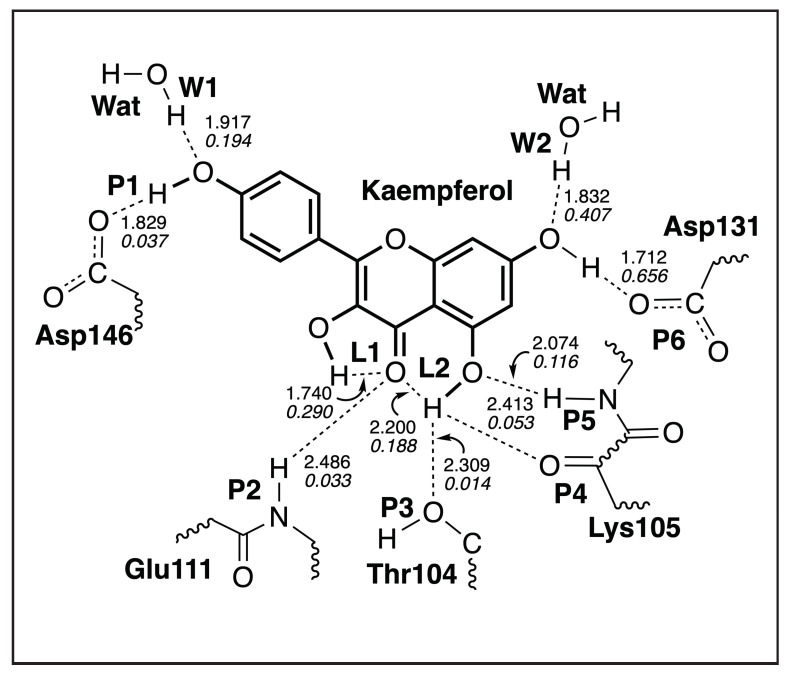
Hydrogen bonds in the Kaempferol-NS5 complex (dashed lines). Bond lengths are shown in the normal font, BSO values are shown in the italic font. ωB97X-D/6-31G(d,p)/AMBER level of theory.

**Figure 12:**
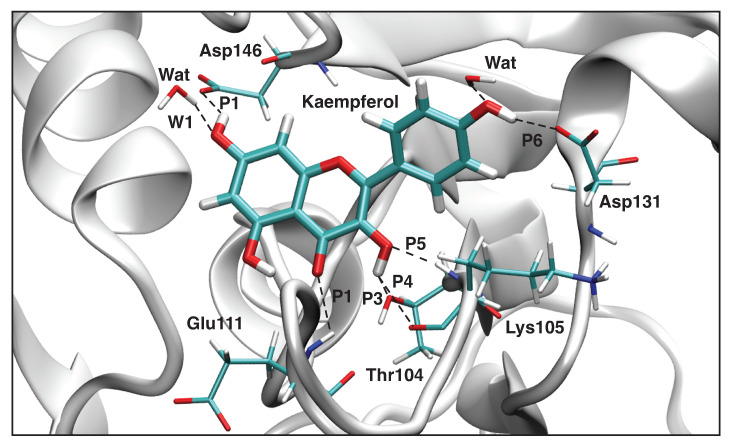
Optimized geometry of the Kaempferol-NS5 complex. The QM part includes Kaempferol, indicated by bold sticks; the MM part includes the NS5 protein, water molecules, and counter ions (for further details, see Supporting Information). There are ten hydrogen bonds (dashed lines) between Kaempferol and the Asp146, Asp131, Lys105, Thr104, and Glu111 residues (thin sticks) of NS5 between Kaempferaol and the two water molecules and two intramolecular hydrogen bonds; see also Figure 11. (Number of QM atoms: 31, total number of atoms: 14,337). ωB97X-D/6-31G(d,p)/AMBER level of theory.

Table 5 shows the hydrogen bond parameters between Kaempferol and NS5 obtained from the QM/MM calculations; a sketch of these hydrogen bonds is shown in Figure 11. Figure 12 presents the picture of the optimal geometry of the ligand in the active site pocket. In our calculations, we observed six hydrogen bonds between Kaempferol and NS5 (**P1**–**P6**), two hydrogen bonds which are formed between the ligand and water molecules (**W1** and **W2**), and two intramolecular hydrogen bonds in the ligand (**L1** and **L2**). According to our calculations, the strongest hydrogen bond between the ligand and the amino acids of the ligand pocket is **P6**, which is formed between the ligand hydroxyl group and the Asp131 side chain, where the hydroxyl group acts as a hydrogen bond donor (BSO = 0.410 and d = 1.712 Å). The weakest hydrogen bond with the amino acids is **P3** (BSO = 0.116 and d = 2.309 Å), which is formed between the ligand hydroxyl group and the hydroxyl group of the Thr104 side chain, where the ligand acts as a hydrogen bond donor. The weakness of this hydrogen bond is related to the fact that this hydroxyl group of the ligand is also involved in three other hydrogen bonds as well—namely, **P4**, which is between the ligand hydroxyl group and the carbonyl group from the backbone of Lys105; **P5**, which is between the ligand hydroxyl group and the amino group in the backbone of Lys105; and the intramolecular hydrogen bond **L2**, which is between the hydroxyl group and the carboxyl group inside the ligand. The strongest hydrogen bond between the ligand and water molecule is **W2**, which is formed between the hydroxyl group of the ligand with a water molecule acting as a hydrogen bond donor (BSO = 0.350 and d = 1.832 Å). Generally, we can conclude that the strongest interaction between the ligand and the NS5 protein pocket involves the hydroxyl group in the 4H-chromen-4-one part of the ligand, which forms the **P6** and **W2** hydrogen bonds.

#### 3.4.3. Quercetin-NS3

**Figure 13:**
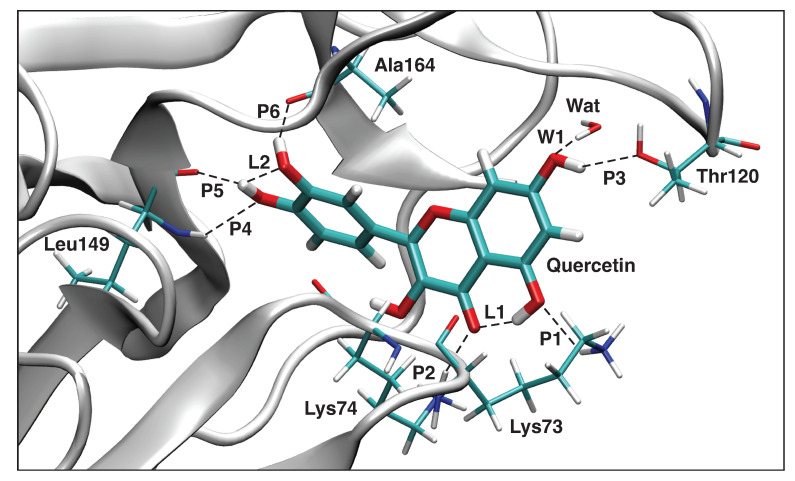
The optimized geometry of the Quercetin-NS3 complex. The QM part includes Quercetin, indicated by bold sticks; the MM part includes the NS3 protein, water molecules, and counter ions (for further details, see Supporting Information). There are nine hydrogen bonds (dashed lines) between Quercetin and the Ala164, Thr120, Lys73, Lys74, and Leu149 residues (thin sticks) of NS3, between Quercetin and a water molecule, and between two intramolecular hydrogen bonds; see also Figure 14. (Number of QM atoms: 32, total number of atoms: 3051). ωB97X-D/6-31G(d,p)/AMBER level of theory.

The local mode parameters of the hydrogen bonds between Quercetin and the NS3 protein pocket from the QM/MM calculations are presented in Table 6, and Figure 13 presents the picture of the optimal geometry of the ligand in the active site pocket. We observe in the calculations six hydrogen bonds between the ligand and the protein amino acids **P1**–**P6**, two hydrogen intramolecular bonds **L1**–**L2**, and one hydrogen bond with a water molecule **W1**. According to our LMA analysis, the strongest hydrogen bond **P5** (k${}^{a}$ = 0.470 mDyn/Å) is formed between the hydroxyl group of the ligand and the carbonyl group of Leu149. That hydrogen bond has a bifurcated character, where the hydrogen atom of the ligand hydroxyl group forms also the relatively strong intramolecular hydrogen bond with the oxygen atom of the other ligand hydroxyl group **L2** (k${}^{a}$ = 0.216 mDyn/Å). In that part of the ligand, there is also a second strongest hydrogen bond **P6** (k${}^{a}$ = 0.408 mDyn/Å), formed between the hydroxyl group of the ligand and Ala164. Both the strongest hydrogen bonds of the Quercetin and the NS3 protein pocket are hydrogen atom donors. The same part of the ligand is additionally stabilized in the pocket by a relatively weak hydrogen bond **P4** (k${}^{a}$ = 0.049 mDyn/Å), formed between the oxygen atom of the ligand hydroxyl group and the hydrogen atom of Leu149, where the side chain of this amino acid acts as a hydrogen atom donor. The other part of the ligand forms two hydrogen bonds **P1** (k${}^{a}$ = 0.042 mDyn/Å) and **P2** (k${}^{a}$ = 0.289 mDyn/Å) between two oxygen atoms of the ligand and the hydrogen atoms of Lys73 and Lys74, respectively. The additional stabilization of that part of the ligand in the protein pocket is achieved by two hydrogen bonds between the hydroxyl group of the ligand and the oxygen atom of Thr120 **P3** (k${}^{a}$ = 0.116 mDyn/Å), as well as between the ligand hydroxyl group and the hydrogen atom of water Wat **P3** (k${}^{a}$ = 0.308 mDyn/Å).

#### 3.4.4. Quercetin-NS5

**Figure 15:**
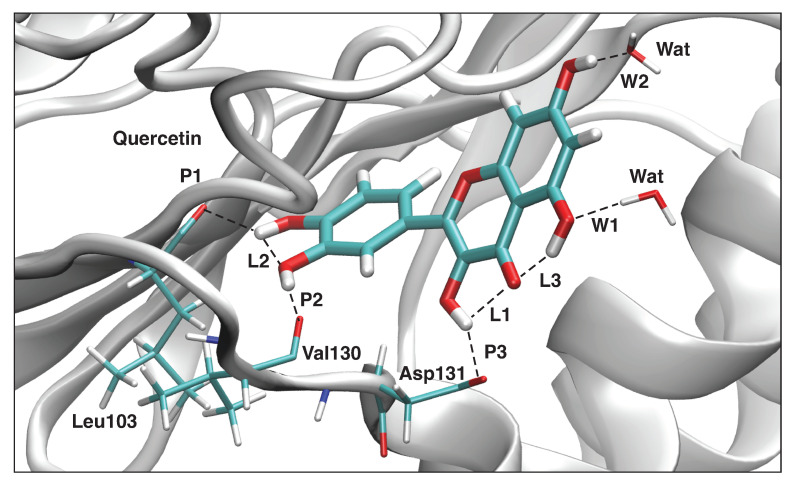
Optimized geometry of the Quercetin-NS5 complex. The QM part includes Quercetin, indicated by bold sticks; the MM part includes the NS5 protein, water molecules, and counter ions (for further details, see Supporting Information). There are eight hydrogen bonds (dashed lines) between Quercetin and the Leu103, Val130, and Asp131 residues (thin sticks) of NS5, between Quercetin and the two water molecules, and between three intramolecular hydrogen bonds; see also Figure 16. (Number of QM atoms: 32, total number of atoms: 14,116). ωB97X-D/6-31G(d,p)/AMBER level of theory.

The results of our LMA analysis based on the QM/MM calculations of the Quercetin and the NS5 protein pocket are presented in Table 7, and the picture of the optimal geometry of the ligand in the protein pocket is shown in Figure 15. According to our results there are three hydrogen bonds between the ligand and the protein amino acids **P1**–**P3**, three intramolecular hydrogen bonds **L1**–**L3**, and two hydrogen bonds with water molecules **W1**–**W2**. The strongest hydrogen bond **P3** (k${}^{a}$ = 0.500 mDyn/Å) is formed between the hydrogen atom of the ligand hydroxyl group and the oxygen atom of Asp131. This hydrogen bond has a bifurcated character because the hydrogen atom of that ligand hydroxyl group forms also a relatively strong intramolecular hydrogen bond **L1** (k${}^{a}$ = 0.227 mDyn/Å). The second strongest hydrogen bond is between the hydrogen atom of the other ligand hydroxyl group and the oxygen atom of Val130 **P2** (k${}^{a}$ = 0.303 mDyn/Å), where the oxygen atom of the ligand hydroxyl group is also involved in the other intramolecular hydrogen bond **L2** (k${}^{a}$ = 0.221 mDyn/Å). The hydrogen atom of the intramolecular hydrogen bond **L2** is also involved in the hydrogen bond **P1** (k${}^{a}$ = 0.212 mDyn/Å) making the bifurcated character of this hydrogen bond. The additional stabilization of the ligand in the protein pocket is due to two hydrogen bonds with two water molecules. The hydrogen bond **W1** (k${}^{a}$ = 0.205 mDyn/Å) is formed between the oxygen atom of the ligand hydroxyl group and the water hydrogen atom, while the hydrogen bond **W2** (k${}^{a}$ = 0.387 mDyn/Å) is formed between the hydrogen atom of the other ligand hydroxyl group and the water oxygen atom.

Our final compounds (Kaempferol and Quercetin) showed exceptional results concerning MD, MMGBSA-free-energy calculation, ADME, QM/MM, and LMA analyses, confirming them as possible scaffolds for the DENV 2 virus. We would like to point out that Kaempferol and Quercetin may not be fully recognized as potent drug candidates in the current literature. For example, based on HEK293T/17 and BHK-21 assays, Care et al. [123] suggested that Kaempferol may enhance infection of the DENV. However, a caveat is appropriate; the conclusions drawn are, in general, sensitive to the choice of statistical methods and data sets utilized for the genotoxicity experiments, and therefore, demand multiple assay routes [124]. Apart from cell-based assays, other issues may come into play, such as activity, physiochemical properties, metabolic stability, toxicology, etc. [125]. Therefore, the Pharma world utilizes, in general, a variety of techniques complementing each other to optimize for each property [126,127,128]. Thus, scavenging for a scaffold is the key point in triggering optimization routes that can later be adopted for the full realization of a potent drug. As an example, Kaempferol derivatives including Kaempferol-3-O-hexose, Kaempferitrin, Kaempferol-3-O-hexose [129], Kaempferol-3,7-diglucoside [130], and Kaempferol 3-O-β-rutinoside [131] were reported as anti-viral agents against dengue in in vitro and in vivo studies. Therapeutic mechanisms, potential host factors [132], and effects on the intracellular DENV virus replication [133] of Quercetin were experimentally identified, proving the anti-dengue capacity of this papaya leaf extract, thus, strongly supporting our study.

## 4. Conclusions and Outlook

In this study, we identified three potential anti-viral compounds (Quercetin, Kaempferol, and Chlorogenic acid) from papaya leaf extracts against the dengue virus. As evidenced by the results of classical docking, MD simulations, ADME, and interaction analysis, our final compounds show inhibitory potential for the NS3 and NS5 proteins in DENV2.

MM/GBSA-free-energy calculations revealed the highest binding free energy for Kaempferol. The RMSD analysis of the MD simulations confirmed the stability of the three NS3-ligand and the three NS5-ligand complexes, with a maximum deviation below 2 Å. ADME, drug-likeness, and drug target studies using the SwissADME and SwissTargetPrediction recommended Quercetin and Kaempferol as orally bioavailable, with good gastrointestinal absorption and better pharmacokinetic properties with low blood-brain barrier permeability.

Therefore, for the Kaempferol-NS3, Quercetin-NS3, Kaempferol-NS5, and Quercetin-NS5 complexes, QM/MM geometry and frequency calculations were performed followed by the local mode analysis developed in our group to quantify ligand-NS hydrogen bonding. The QM/MM and LMA analysis of Kaempferol revealed 8 (NS3) and 10 (NS5), and Quercetin showed 9 (NS3) and 8 (NS5) HBs. The strongest HB between Kaempferol and the amino acids of the protein pocket is formed between the backbone of Ala164 (BSO = 0.237 and d = 1.880 Å) for NS5 and the side chain of Asp131 (BSO = 0.410 and d = 1.712 Å) for NS3, as well as Leu149 (BSO = 0.367 and d = 1.790 Å) for NS3 and Asp131 (BSO = 0.375 and d = 1.752 Å) for NS5 in the Quercetin; therefore, quantifying, for the first time, the strong NS3 and NS5 protein binding ability of Kaempferol and Quercetin, thus, qualifying this papaya extract as a strong therapeutic candidate for the treatment of DENV infection. Overall, this in silico study, following our new protocol stretching from simple classical docking to the high-accuracy QM/MM calculation developed in this work, highlights the value of the structure-based drug design to efficiently identify new drug candidates for subsequent in vitro and in vivo studies, and as such, helping to reduce the cost and time needed for the development of a new drug.

The NS5 protein is the major target for new anti-dengue drug therapies due to its involvement in RNA replications of Flaviviruses. This protein is observed in various members of the Flavivirus genus, including, but not limited to, the West Nile virus (WNV), Zika virus (ZIKV), Japanese encephalitis virus (JEV), tick-borne encephalitis virus (TBEV), and yellow fever virus (YFV). Thus, the information gained in this study by targeting NS5 to overcome dengue virus infections can be extended to other species of the Flavivirus genus, impacting a wider range of antiviral treatments. Work is in progress to apply our new protocol to other potential anti-viral compounds being isolated from natural products. 

## Figures and Tables

**Figure 14:**
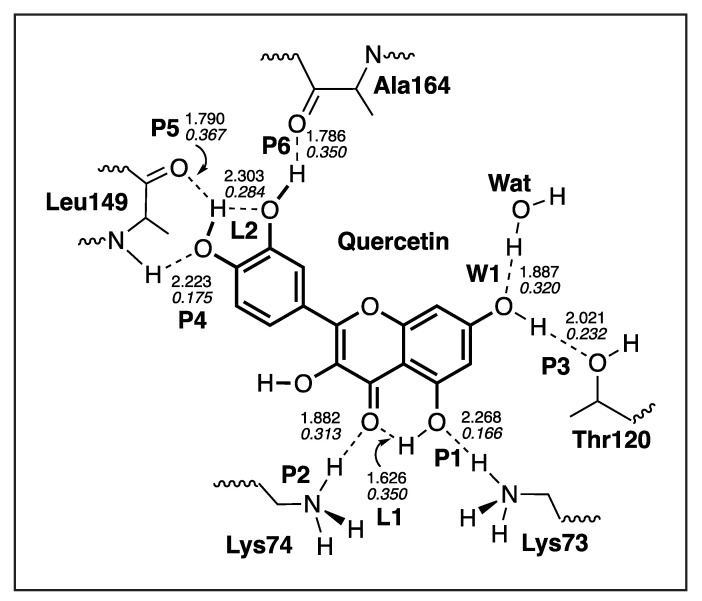
Hydrogen bonds in the Quercetin-NS3 complex (dashed lines). Bond lengths are shown in the normal font, BSO values are shown in the italic font. ωB97X-D/6-31G(d,p)/AMBER level of theory.

**Figure 16:**
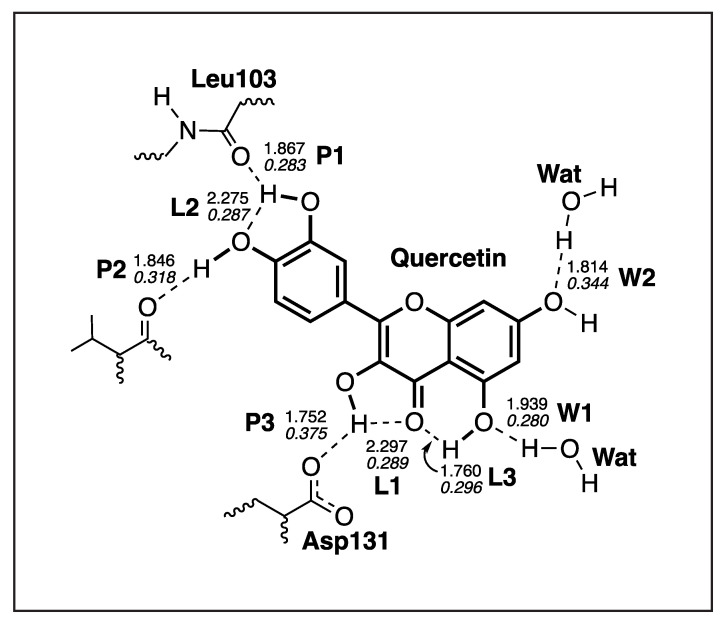
Hydrogen bonds in the Quercetin-NS5 complex (dashed lines). Bond lengths are shown in the normal font, BSO values are shown in the italic font. ωB97X-D/6-31G(d,p)/AMBER level of theory.

**Table 1:** Smina binding affinities of the seven compounds with regard to the NS3 and NS5 proteins.

Compounds	NS3 (kcal/mol)	NS5 (kcal/mol)
**Quercetin**	**−9.2**	**−10.1**
**Kaempferol**	**−8.9**	**−9.6**
**Chlorogenic acid**	**−8.6**	**−9.2**
Caffeic acid	−6.8	−6.5
p-Coumaric acid	−6.2	−6.2
Protocatechuic acid	−6.4	−6.1
5,7-Dimethoxycoumarin	−6.2	−6.0

**Table 2:** Thermodynamic binding free energy (average) for PLE compounds from MM/GBSA with NS3 and NS5 proteins.

	Components (kcal/mol)					
**Protein**	**Compounds**	**ΔE_vdW_**	**ΔE_elec_**	**ΔG_gas_**	**ΔG_solv_**	**ΔG_bind_**
	Quercetin	−33.41 ± 0.20	−29.70 ± 0.45	−63.15 ± 0.57	43.06 ± 0.82	−20.09 ± 0.18
NS3	Kaempferol	−36.04 ± 0.15	−19.32 ± 0.39	−55.36 ± 0.38	30.33 ± 0.32	−25.03 ± 0.15
	Chlorogenic acid	−22.75 ± 0.25	−53.05 ± 0.98	−75.79 ± 0.95	63.59 ± 0.86	−12.19 ± 0.21
	Quercetin	−27.71 ± 0.18	−23.77 ± 0.49	−51.49 ± 0.50	33.30 ± 0.43	−18.19 ± 0.16
NS5	Kaempferol	−34.37 ± 0.26	−27.02 ± 0.39	−45.92 ± 0.47	39.70 ± 0.41	−25.77 ± 0.17
	Chlorogenic acid	−27.90 ± 0.22	−45.97 ± 0.73	−72.87 ± 0.80	53.50 ± 0.65	−19.37 ± 0.24

**Table 3:** Predicted ADME parameters, drug-likeness, pharmacokinetic, and physicochemical properties of CP-leaf compounds using the SwissADME server.

Compounds	Molecular Formula	Molecular Weight (g/mol)	Lipophilicity (iLOGP)	Water Solubility	GIT Absorption	BBB Permeability	Bioavailability Score	Synthetic Accessibility	Drug-Likeness (Lipinski)
Quercetin	C_15_H_10_O_7_	302.24	1.63	Soluble	High	No	0.55	3.23	Yes
Kaempferol	C_15_H_10_O_6_	286.24	1.70	Soluble	High	No	0.55	3.14	Yes
Chlorogenic acid	C_16_H_18_O_9_	354.31	0.96	Soluble	Low	No	0.11	4.16	Yes(1)

**Table 4:** The local mode parameters of the hydrogen bonds between Kaempferol and NS3 from the QM/MM calculations, the ωB97X-D/6-31G(d,p)/AMBER level of theory for the calculation in the protein, and the ωB97X-D/6-31G(d,p) level of theory in the gas phase.

H-Bond	d	k${}^{a}$	$\omega^{a}$	BSO
	Å	mDyn/Å	cm${}^{-1}$	
**L1**	1.694	0.316	752	0.322
**P1**	2.094	0.066	344	0.193
**P2**	1.880	0.124	471	0.237
**P3**	2.212	0.063	336	0.190
**P4**	2.508	0.015	163	0.118
**W1**	1.838	0.374	818	0.341
**W2**	1.912	0.240	655	0.294
**W3**	1.809	0.490	937	0.372
**WD ^1^**	1.926	0.225	635	0.288

^1^ H_2_O⋯HOH water dimer in the gas phase.

**Table 5:** The local mode parameters of the hydrogen bonds between Kaempferol and NS5 from the QM/MM calculations, the ωB97X-D/6-31G(d,p)/AMBER level of theory for the calculation in the protein, and the ωB97X-D/6-31G(d,p) level of theory in the gas phase.

H-Bond	d	k${}^{a}$	$\omega^{a}$	BSO
	Å	mDyn/Å	cm${}^{-1}$	
**L1**	1.740	0.290	720	0.313
**L2**	2.200	0.188	579	0.272
**P1**	1.829	0.037	258	0.159
**P2**	2.486	0.033	243	0.153
**P3**	2.309	0.014	158	0.116
**P4**	2.413	0.053	307	0.179
**P5**	2.074	0.116	455	0.232
**P6**	1.712	0.656	1084	0.410
**W1**	1.917	0.194	589	0.275
**W2**	1.832	0.407	853	0.350
**WD ^1^**	1.926	0.225	635	0.288

^1^ H_2_O⋯HOH water dimer in the gas phase.

**Table 6:** The local mode parameters of the hydrogen bonds between Quercetin and NS3 from the QM/MM calculations, the ωB97X-D/6-31G(d,p)/AMBER level of theory for the calculation in the protein, and the ωB97X-D/6-31G(d,p) level of theory in the gas phase.

H-Bond	d	k${}^{a}$	$\omega^{a}$	BSO
	Å	mDyn/Å	cm${}^{-1}$	
**P1**	2.268	0.042	276	0.166
**P2**	1.882	0.289	719	0.313
**P3**	2.021	0.116	455	0.232
**P4**	2.223	0.049	297	0.175
**P5**	1.790	0.470	917	0.367
**P6**	1.786	0.408	854	0.350
**L1**	1.626	0.407	854	0.350
**L2**	2.303	0.216	622	0.284
**W1**	1.887	0.308	743	0.320
**WD ^1^**	1.926	0.225	635	0.288

^1^ H_2_O⋯HOH water dimer in the gas phase.

**Table 7:** The local mode parameters of the hydrogen bonds between Quercetin and NS5 from the QM/MM calculations, ωB97X-D/6-31G(d,p)/AMBER level of theory for the calculation in the protein, and ωB97X-D/6-31G(d,p) level of theory in the gas phase.

H-Bond	d	k${}^{a}$	$\omega^{a}$	BSO
	Å	mDyn/Å	cm${}^{-1}$	
**P1**	1.867	0.212	616	0.283
**P2**	1.846	0.303	737	0.318
**P3**	1.752	0.500	946	0.375
**L1**	2.297	0.227	637	0.289
**L2**	2.275	0.221	629	0.287
**L3**	1.760	0.245	663	0.296
**W1**	1.939	0.205	606	0.280
**W2**	1.814	0.387	832	0.344
**WD ^1^**	1.926	0.225	635	0.288

^1^ H_2_O⋯HOH water dimer in the gas phase.

## Data Availability

All data supporting the results of this work are presented in tables and figure of the manuscript.

## Supplementary Materials

The following are available online at https://www.mdpi.com/article/10.3390/ijms232012310/s1.
