# Supplementary material for: Papaya Leaf Extracts as Potential Dengue Treatment: An In-Silico Study
Source: Int J Mol Sci. 2022 Oct 14;23(20):12310. doi: 10.3390/ijms232012310 (PMC9610845; doi:10.3390/ijms232012310)
Supplement: Supplementary file 1 [file ijms-23-12310-s001.zip › ijms-1860267-supplementary.pdf]

International Journal of
*Molecular Sciences*

MDPI

*Article*

# Supporting Materials: Papaya Leaf Extracts as Potential Dengue Treatment: An In-Silico Study

**Ayesh Madushanka [1], Niraj Verma [1], Marek Freindorf [1] and Elfi Kraka [1]***

[1]Current address: Southern Methodist University; Department of Chemistry; 3215 Daniel Avenue; P.O. Box 750314; Dallas, Texas 75275.
* Correspondence: ekraka@smu.edu

**Citation:** Madushanka, A.; Verma, N.; Freindorf, M.; Kraka, E. Papaya Leaf Extracts as Potential Dengue Treatment: An In-Silico Study. *Int. J. Mol. Sci.* **2022**, *23*, 12310. https://doi.org/10.3390/ijms232012310

Academic Editors: Christo Z. Christov and Tatyana Karabencheva-Christova

Received: 26 July 2022

Accepted: 6 October 2022

Published: 14 October 2022

**Publisher's Note:** MDPI stays neutral with regard to jurisdictional claims in published maps and institutional affil-iations.

**Copyright:** © 2022 by the authors. Submitted to *Journal Not Speci ied* for possible open access publication under the terms and conditions of the Creative Commons Attribution (CC BY) license (https://creativecommons.org/licenses/by/4.0/).

## Contents

**1. Computational Methods** . . . . . . . . . . . . . . . . . . . . . . . . . . . . . . . . **1**
1.1. QM/MM calculations . . . . . . . . . . . . . . . . . . . . . . . . . . . . . . 1

**2. Results and Discussion** . . . . . . . . . . . . . . . . . . . . . . . . . . . . . . . . **2**
2.1. RMSD Analysis . . . . . . . . . . . . . . . . . . . . . . . . . . . . . . . . 2
2.1.1. Second and third MD simulations . . . . . . . . . . . . . . . . . . . . . 2

## 1. Computational Methods

### *1.1. QM/MM calculations*

MM neglects electrons of a molecular system and as such it not able to describe electronic features of chemical bonding and weak chemical interactions in a quantitative way. Depending on the QM method used and the size of the QM part considered QM/MM can provide molecular properties including binding energies with an accuracy in the kcal/mol range. Moreover, electronic embedding in the QM/MM method incorporates important cross-region electrostatic interactions in the QM Hamiltonian, which avoids the approximation of the QM charge distribution by point charges and allows the wave function to be polarized by the charge distribution of the MM region. In this way, non-bonded interactions (HBs) between ligand and protein residues as well HBs with water molecules inside the binding pocket are well described. It also has to be noted that the local mode analysis is based on the entire Hessian involving both the QM and MM atoms. Therefore the local mode force constants of HBs s betweenQM and MM atoms correctly reproduces the strength of these bonds.

The QM/MM calculations of both NS proteins with Kaempferol and Quercitin were performed according to the following protocol: Cartesian coordinates of protein and ligand obtained from the docking procedure were neutralized with 3 $Na^+$ and 11 $Cl^-$ counter-ions, respectively for NS3 and the NS5, based on the electrostatic potential of AMBER. The docking sites of the proteins were surrounded with a sphere of TIP3P water molecules at a radius of 16 Å in order to mimic the aqueous environment. $Zn^{+2}$ and $Mg^{+2}$ cations were removed from the NS5 structure because they were located more than 35 Å away from the ligand. Hydrogen atoms were placed according to standard AMBER rules, and the molecular structures were initially minimized with AMBER on the molecular mechanical level. After minimization, the molecular systems were divided into a QM part, which included Kaempferol or Quercitin and an MM part including the protein, counter-ions, and water molecules. The following QM/MM geometry optimizations were performed using ONIOM with electronic embedding without constraints, followed by vibrational frequency calculations using the $\omega$B97X-D/6-31G(d,p)/AMBER level of theory. The optimized minimum structures of both protein systems were identified as local minima, i.e., no imaginary frequencies were observed during the normal mode analysis. Relative

bond strength orders BSO for HBs in Kaempferol-NS3/NS5 and Quercetin-NS3/NS5 complexes calculated from local mode HB force constants via a power relationship utilizing the generalized Badger rule , are graphically presented in Figure S1.

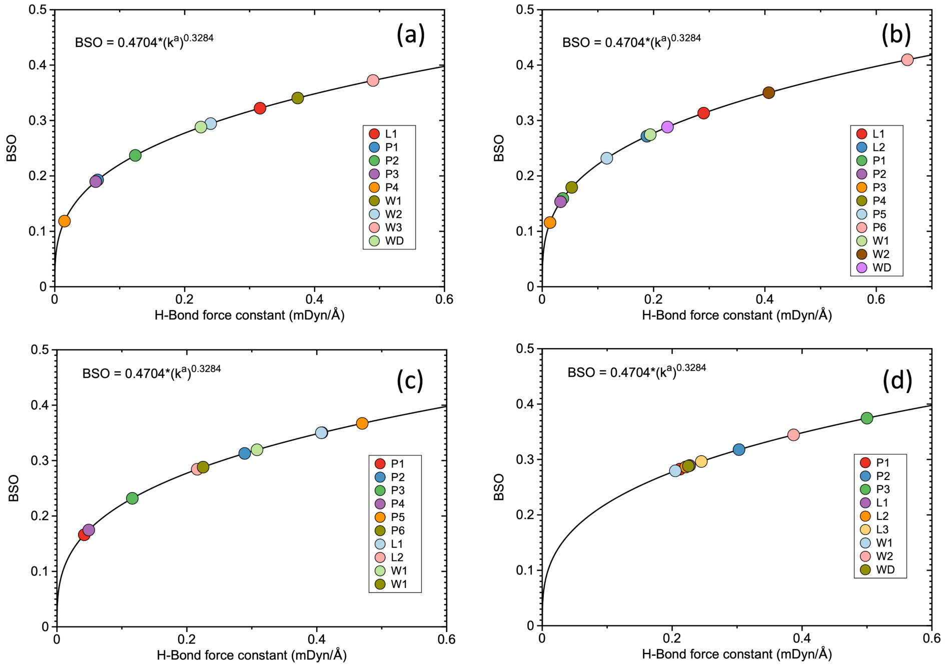


**Figure S1.** Power relationship between the local mode force constant and BSO for hydrogen bond between (a) Kaempferol and NS3, (b) Kaempferol and NS5, (c) Quercetin and NS3 and (d) and NS5. The $\omega$B97X-D/6-31G(d,p)/AMBER level of theory.

## 2. Results and Discussion

### *2.1. RMSD Analysis*

#### 2.1.1. Second and third MD simulations

RMSD of second and third MD simulations are shown in Figure S2 and S3, respectively. The NS5 protein complexes and unbound protein are deviated only between 2 Å distance in all three simulations and showed the same behavior. However, fluctuations were visible over the course of simulation. On the other hand, the second and third RMSDs of the NS3 protein showed distinct deviations from the first s imulation. Nevertheless, all the simulations deviate 2 - 4 Å range showing the stability of the ligands inside the binding pocket. Furthermore, all systems obtained equilibration at around 10 ns and the systems remained stable until 20 ns. In the case of the second simulation, Kaempferol and Chlorogenic acid showed sudden increases resulting to carry out another RMSD analysis for the NS3 binding pockets (residues between 5 Å distance from the ligand). RMSD analysis of the binding pocket is shown in Figure S4 and depicted the stability of the protein-ligand complexes showing the equilibration after the 10 ns except for Chlorogenic acid. We have not taken Chlorogenic acid for the QMMM and LMA analysis. However, to identify the confirmational changes, long MD simulations are needed.

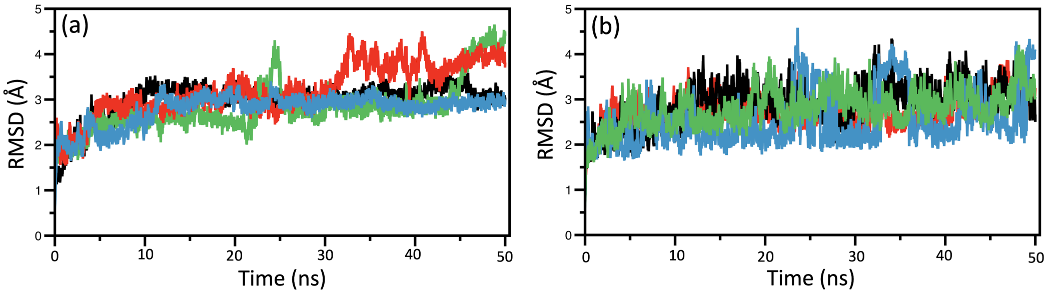


**Figure S2.** RMSD analysis of second MD simulation for (a) NS3 and (b) NS5 receptors. by NS5 :Black - unbound protein, Blue - Quercetin, Red - Kaempferol and Green - Chlorogenic acid.

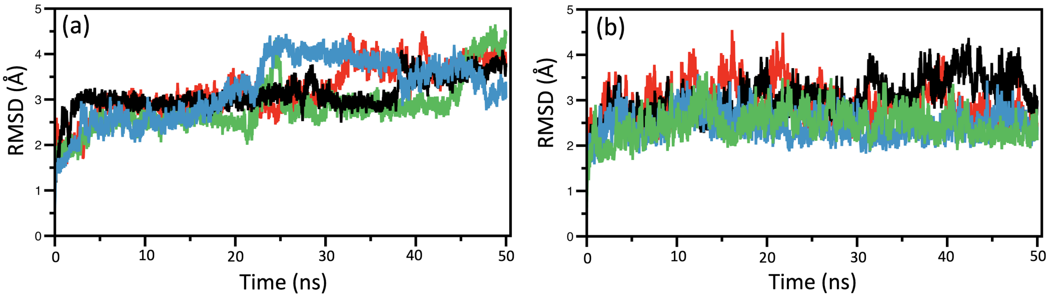


**Figure S3.** RMSD analysis of third MD simulation for (a) NS3 and (b) NS5 receptors. by Black - unbound protein, Blue - Quercetin, Red - Kaempferol and Green - Chlorogenic acid.

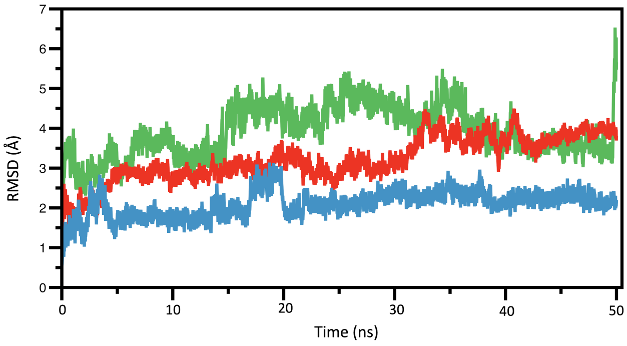


**Figure S4.** RMSD analysis of the pocket (second MD simulation) between 5 Å for the NS3 receptor. by Blue - Quercetin, Red - Kaempferol and Green - Chlorogenic acid.
